# Assessing the Angiogenic Potential of Poly(ε-Caprolactone) PCL/Bioactive Glass Composites in a Co-Culture Model of ASCs and HMEC-1

**DOI:** 10.3390/biomedicines14051109

**Published:** 2026-05-14

**Authors:** Clarissa Orrico, Ilaria Roato, Alessandro Mosca Balma, Sara Meinardi, Giacomo Baima, Tullio Genova, Marta Miola, Enrica Verné, Federico Mussano

**Affiliations:** 1 Bone and Dental Bioengineering Laboratory, CIR Dental School, Department of Surgical Sciences, University of Turin, 10126 Turin, Italy; clarissa.orrico@unito.it (C.O.); ilaria.roato@unito.it (I.R.); alessandro.moscabalma@unito.it (A.M.B.); sara.meinardi@unito.it (S.M.); 2 Department of Mechanical and Aerospace Engineering, Politecnico di Torino, Corso Duca degli Abruzzi 24, 10129 Turin, Italy; 3 Periodontology Unit, Department of Surgical Sciences, CIR Dental School, University of Turin, 10126 Turin, Italy; giacomo.baima@unito.it; 4 Department of Life Sciences and Systems Biology, University of Torino, Via Accademia Albertina 13, 10123 Torino, Italy; tullio.genova@unito.it; 5 Department of Applied Science and Technology, Politecnico di Torino, Corso Duca degli Abruzzi 24, 10129 Turin, Italy; marta.miola@polito.it (M.M.); enrica.verne@polito.it (E.V.)

**Keywords:** poly(ε-caprolactone), bioactive glass, fused deposition modeling (FDM), mesenchymal stromal/stem cells, endothelial cells, pre-osteoblast/endothelial cells co-culture, copper-doped, early cell response, angiogenic potential, in vitro tubulogenesis assay

## Abstract

**Background/Objectives**: An ideal bone scaffold should promote bone cell growth and functional vascularization, hence the importance of imbuing biomaterials with pro-angiogenic cues. In this work, silica-based bioactive glasses, either pristine (SBA3) or doped with copper (SBA3_Cu), were embedded in poly(ε-caprolactone) (PCL), which was also used as a control. **Methods**: In vitro co-cultures of adipose-derived mesenchymal stem/stromal cells (ASCs) and human microvascular endothelial cells (HMEC-1s) were kept in α-MEM, MCDB131, and EndoGRO media to test the biomaterials. The co-cultures were visualized by immunofluorescence and SEM, while flow cytometry was performed to characterize cellular immunophenotype. The angiogenic potential was evaluated using conditioned media of co-cultures to perform a tubulogenesis assay and VEGF-A quantification. **Results**: Immunophenotypic analysis showed a significant decrease in the endothelial CD31+ cellular subset, whereas the OB-like cellular subset expressing CD105, CD73, CD90, and ALP increased in all culture media over time. In α-MEM, HMEC-1s were unable to form a capillary network independent of the substrates. A more organized network was visible when co-cultures were plated on PCL, in MCDB131 and EndoGRO, or if they were kept in EndoGRO on PCL/SBA3_Cu. The VEGF-A concentrations were similar in the conditioned media from co-cultures grown on PCL/SBA_Cu, in EndoGRO, and on PCL and PCL/SBA3, in MCDB131. **Conclusions**: The presence of copper did not promote the angiogenic potential of HMEC-1, likely due to the low concentration of released copper ions and the predominant osteoinductive effect of the other ions released by the bioglass. A re-evaluation of formulation and structure of bioglass scaffold could enhance the angiogenic potential.

## 1. Introduction

Alloplastic bone grafts mimicking the composition and architecture of the osseous extracellular matrix are the long-term goal of the research endeavors aiming to treat bone defects while overcoming the pitfalls of autologous, allogenic, and xenogenic bone grafts [1,2]. An ideal bone scaffold should provide structural and physiological support to the healing cells, guiding tissue regeneration [3]. One of the critical aspects to be examined when designing bone scaffolds is to support cellular adhesion and proliferation, respecting the architectural peculiarity of the defect to be restored. In this regard, bone-forming pre-osteoblasts/osteoblasts (OBs) are just as critical as endothelial cells in enabling the development of a vascularized network [4]. Hence, cellular models that entail the co-culture of both these cell types have gained increasing consideration to better reproduce the biological complexity [5].

Owing to their osteoinductive capability [6], bioactive glasses (BGs) have been thoroughly investigated in the bone tissue engineering (BTE) field, during the past decades [7,8]. However, successful neovascularization (the sprouting of blood vessels) in artificial constructs seems challenging [9], which is consistent with the limited translation they have had in the clinical treatment of periodontal defects [10]. Under this perspective, as Cu^2+^ is known to exert a pro-angiogenic effect by activating growth factors, like vascular endothelial growth factor (VEGF) and platelet-derived growth factor (PDGF) [11,12,13], Cu-doped BGs have been considered a promising tool to enhance the vascularization in bone tissue regeneration. Consequently, the local delivery of Cu^2+^ from Cu-doped BGs was investigated, with encouraging results, in various experimental conditions such as mesoporous BGs [14], functional coatings of BG nanoparticles [15], and electrospun nanofibers of BGs [16], highlighting a dose- and exposure-duration-dependent effect in balancing the efficacy and cytotoxicity of the released Cu^2+^ ions. However, in the above-mentioned studies, the actual role of copper-doped BG in angiogenesis and the related mechanisms are only partially explained. Furthermore, the mechanical properties of bulk BGs are often poor and make it necessary to use processing technologies, which are sometimes very complex, to produce pure BG-based bone scaffolds. In contrast, powdered BGs are very suitable as fillers within polymer matrices.

Polycaprolactone (PCL), a synthetic and bioresorbable aliphatic polyester, has emerged as a promising polymer for BTE applications [17]. PCL can be easily processed with 3D printing techniques [18,19], which offer an optimal control of the scaffold architecture, including pore size, distribution, and interconnectivity [20]. Recently, fused deposition modeling (FDM) has been used for manufacturing PCL-based composites charged with hydroxyapatite (HA) and β-tricalcium phosphate (β-TCP) [21], or β-TCP alone [22,23], as well as BGs [24].

In the current study, pneumatic FDM was employed to prepare two PCL/BG composites characterized by the presence of a silica-based bioactive glass (SBA3) and its copper-doped formulation (SBA3_Cu). The authors studied whether these two PCL/BGs compounds, along with neat PCL as a control, could promote angiogenesis in vitro, in a co-culture of adipose tissue-derived mesenchymal stem/stromal cells, named ASC52telo (ASCs) and Human Microvascular Endothelial Cells (HMEC-1), representing, respectively, pre-osteoblast and endothelial cells for tissue formation in in vitro models.

## 2. Materials and Methods

### 2.1. Sample Preparation

A thermoplastic pneumatic printhead mounted on a BIO X 3D bioprinter (CELLINK Bico Group, Gothenburg, Sweden) was used to fabricate different ester-terminated poly (ε-caprolactone) (PCL) (CELLINK PCL TP-60505, Bico Group, Gothenburg, Sweden) based samples. Two different formulations of the same bioactive glass, SBA3 and SBA3_Cu, were adopted for the compounds. SBA3 glass powders (composition 48% SiO_2_, 26% Na_2_O, 22% CaO, 3% P_2_O_5_,0.43% B_2_O_3_, 0.57% Al_2_O_3_ mol%) were produced by a melting and quenching technique, followed by ball milling and sieving processes, to obtain a granulometry <20 μm, as previously reported [25]. For the copper-doped formulation (SBA3_Cu), the obtained powders were subjected to an ion-exchange process in a 0.01 M copper acetate solution for 1 h at 37 °C [25]. The compounds were obtained by loading the polymer, respectively, with SBA3 or SBA3_Cu, at 10 wt.%, through solvent casting with Chloroform (CHCl_3_, CARLO ERBA Reagents s.r.l., Cornaredo, Italy), as previously published by the authors’ group [24]. The two compounds, henceforth named PCL/SBA3 and PCL/SBA3_Cu, were compared to the neat PCL (PCL), used as a control for the experiments. The adopted printing parameters were set to maintain a 30 °C print-bed temperature with the internal chamber fan activated, a printhead temperature of 115 °C, and a speed of 2 mm/s. The compounds were extruded by an air pressure flow of 190 kPa through a nozzle of 0.4 mm in diameter. All the compounds are reported in Table 1. Square-based planar samples were printed directly onto the glass surface of a Petri dish to ensure a smooth surface result. To fit the dimensions of a 96-well culture plate, the planar samples were cut using a 6 mm biopsy punch.

### 2.2. Cell Culture

The ASC52telo cell line, hTERT immortalized adipose-derived mesenchymal stem cells (ASCs) (ATCC, Manassas, VA, USA), and human microvascular endothelial cells (HMEC-1s) (CLS, Cell Lines ServiceGmbH, Eppelheim, Germany) were used to perform all the experiments on the different printed samples. To expand them, ASCs and HMEC-1 were grown, respectively, in ASC medium (Mesenchymal Stem Cell Basal Medium (ATCC PCS-500-030) with the Mesenchymal Stem Cell Growth Kit (ATCC PCS-500-040)), and in MCDB131 medium (Life Technologies, Carlsbad, CA, USA) with 1% L-Glutamine, 10% fetal bovine serum (FBS), 1% Penicillin (100 U/mL)-Streptomycin (100 μg/mL) (Life Technologies, Carlsbad, CA, USA), 1 µg/mL Hydrocortisone and 10 ng/mL EGF (Merck KGaA, Darmstadt, Germany), at 37 °C in a 5% CO_2_ atmosphere.

For the experiments, ASCs and HMEC-1 were seeded (1:3 ratio) onto a standard multi-well for cell culture and the different 2D samples in α-MEM with 10% fetal bovine serum (FBS), 1% Penicillin (100 U/mL)-Streptomycin (100 μg/mL) (Life Technologies, Carlsbad, CA, USA), or in MCDB131 (growth medium for HMEC-1), or in EndoGRO™-MV-VEGF (henceforth abbreviated as EndoGRO, composed as follows: EndoGRO™ Basal Medium supplemented with 5% FBS, 5 ng/mL rhVEGF, 5 ng/mL rhEGF, 5 ng/mL rhFGF, 15 ng/mL rhIGF-1, 1.0 μg/mL hydrocortisone hemi-succinate, 0.75 U/mL heparin sulfate, 10 mM L glutamine, and 50 μg/mL ascorbic acid according to manufacturer’s instructions) (Merck KGaA, Darmstadt, Germany).

### 2.3. Immunofluorescence Staining of HMEC-1 Co-Cultured with ASCs

HMEC-1 (7500 cells/well) and ASCs (2500 cells/well) were cultured on plastic and different surfaces in 96-well culture plates, in α-MEM, MCDB131, and EndoGRO. After 21 days, cells were fixed using 4% paraformaldehyde, rinsed twice with phosphate-buffered saline (PBS), and permeabilized with TBS 1X (Life Technologies, Carlsbad, CA, USA) containing 0.5% Triton X-100 (Biorad, Hercules, CA, USA) for 1 min. After 2 washes with PBS, cells were incubated at room temperature for 30 min with Image-iT™ FX Signal Enhancer (Life Technologies, Carlsbad, CA, USA) to saturate and reduce non-specific signals. Following two washes with PBS, cells were incubated in agitation at room temperature with rabbit monoclonal antibody detecting CD31 (1:300 in TBS 1X + BSA 1% + Tween 0.3%; Abcam, Cambridge, UK) for 1 h and then with secondary antibody goat anti-Rabbit IgG Alexa Fluor 700 (1:1000 in TBS 1X + BSA 1% + Tween 0.3%; Cell Signaling Technology, Danvers, MA, USA) for another 45 min. Next, Alexa Fluor 488 Phalloidin (1:200 TBS 1X + BSA 1% + Tween 0.3%; Cell Signaling Technology, Danvers, MA, USA) staining was performed (45 min of incubation at room temperature) to evidence the cytoskeleton. Lastly, two PBS washes were performed, and nuclei were stained with DAPI (Merck KGaA, Darmstadt, Germany). Images were acquired using a Leica Stellaris 5 confocal microscope (Leica Microsystems, Wetzlar, Germany) at 10× and 20× magnification.

### 2.4. Scanning Electron Microscope (SEM) Analysis

After the printing process, samples were washed in distilled water in a 70 vol.% ethanol/water solution, and absolute ethanol was dried under a chemical hood. A coating with a conductive layer of gold was applied before the acquisitions, which were taken by SEM (Phenom XL G2 Desktop SEM, Thermo Fisher Scientific, Waltham, MA, USA), with 10 kV of source potential in MAP configuration, with a BSD detector and a magnification of 1000× and 5000×. EDX spectra were also performed to assess the surface element composition of each sample. After 21 days of co-culture in different media, images of the biological structures formed on each composition were acquired. The specimens were fixed using 4% paraformaldehyde, rinsed twice with phosphate-buffered saline (PBS), and then dehydrated with progressively higher concentrations of ethanol/water, for 20 min each time, till the 99.9 vol.%, and then dried under a chemical hood. Before SEM analysis, a conductive layer of gold was applied as a coating. The instrument settings were 10 kV of source potential in MAP configuration, with a BSD detector and a magnification of 1550×.

### 2.5. Flow Cytometry Analysis

The immunophenotype of ASCs and HMEC-1 (at 7, 14 and 21 days of co-culture) was assessed through flow cytometry by staining with monoclonal fluorochrome-conjugated antibodies against the following surface antigens: CD105 PE (Life Technologies, Carlsbad, CA, USA), CD73 VioBrightB515, CD90 PerCP, CD31 PE, ALP APC (Miltenyi Biotech, Bergisch Gladbach, Germany). As a control, unstained cells were analyzed. Data were examined on a MACsQuant 10 and computed with MACsQuantify software 3.0.1 (Miltenyi Biotech, Bergisch Gladbach, Germany). The data are presented as percentages of cells expressing specific markers (mean ± SD). Three independent experiments were carried out for each experimental condition.

### 2.6. In Vitro Tubulogenesis Assay

The tubulogenic potential of HMEC- 1 was studied following a previously published protocol [26]. Precisely, HMEC-1 were plated (1 × 10^4^ cells/well) on growth-factor-reduced Matrigel (Corning, New York, NY, USA) in μ-plate 96-well 3D (IBIDI GmBh, Gräfelfing, Germany), with the following conditioned media (diluted 1:2 with fresh medium): PCL α-MEM, PCL/SBA3 α-MEM, PCL/SBA3_Cu α-MEM, PCL MCDB131, PCL/SBA3 MCDB131, PCL/SBA3_Cu MCDB131, PCL EndoGRO, PCL/SBA3 EndoGRO, and PCL/SBA3_Cu EndoGRO. After 5 days of co-culture, supernatants were harvested.

The capability of different copper salts (copper acetate, sulfate, chloride and nitrate) to induce tubulogenesis of HMEC-1 was tested at different concentrations (3, 25, 50 µg/mL).

After 15 h, the cellular network formation in Matrigel was acquired using a Nikon Eclipse Ti-E microscope using a Nikon Plan 10×/0.10 objective (Nikon Instruments, Amsterdam, The Netherlands). Three independent experiments were carried out for each experimental condition. The analysis of the experiments was performed using ImageJ2, version 2.9.0/1.53t, and in particular the ImageJ’s Angiogenesis Analyzer plugin developed by Gilles Carpentier [27]. Different parameters were measured: (a) nodes are pixels with 3 neighbors represented as a circular dot; (b) total master segment length is the sum of segment length encountered in each image; (c) master segments are pieces of three, delimited by two junctions (fused nodes), but not exclusively implicated with one branch; (d) master junctions are links between at least 3 master segments; and (e) isolated segments are binary lines that are not branched.

### 2.7. VEGF-A Quantification in Supernatants

After 5 days, the supernatants from ASCs co-cultured with HMEC-1 onto the different specimens were harvested. In these supernatants, the VEGF-A secretion was assessed by enzyme-linked immunosorbent assay (ELISA), according to the manufacturer’s instructions (Elabscience, Houston, TX, USA).

### 2.8. Statistical Analysis

Unless otherwise specified, statistical analyses were conducted using STATA software (version 18.0; StataCorp, College Station, TX, USA). Data were tested for normality with the Shapiro–Wilk test. Then one-way ANOVA was used to assess variance differences among groups of condition, material and culture media, across various time points, followed by post hoc pairwise comparison with Bonferroni correction to identify which groups showed statistically significant differences. For the tubulogenesis assay, repeated Student’s *t*-tests were applied to analyze shape descriptor differences among groups. A significance threshold of α = 0.05 was used for all tests [24].

## 3. Results

### 3.1. Surface Characterization of the Samples

**Figure 1:**
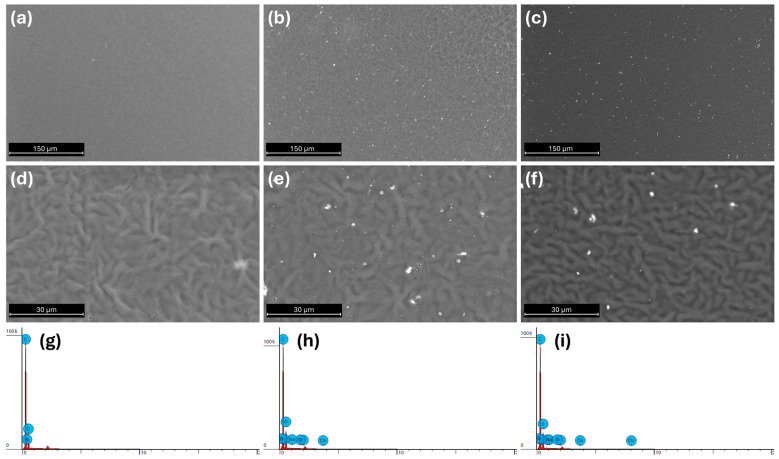
SEM analysis of the surfaces of the materials. SEM images at 1000× and 5000× magnification, respectively, on PCL (**a**,**d**), PCL/SBA3 (**b**,**e**) and PCL/SBA3_Cu (**c**,**f**). EDX spectra of detected elements on the surface of the samples were plotted for PCL (**g**), PCL/SBA3 (**h**) and PCImagL/SBA3_Cu (**i**).

The surface morphology of PCL/SBA3 (Figure 1b,e) and PCL/SBA3_Cu (Figure 1c,f) samples was almost identical, with a homogeneous distribution of the filler inside the polymer without visible bioactive glass aggregates in both the composites. PCL samples (Figure 1a,d) also exhibited a uniform surface with only small pores present, likely caused by the dimensional retraction of the polymer during the cooling process after the print. EDX analysis carried out on the sample surfaces (Figure 1g–i) highlighted the presence of other elements on composite materials different from the PCL ones. Indeed, for the neat PCL, the only acquired elements were carbon, nitrogen, and oxygen, which form the molecular structure of the polymer. In PCL/SBA3 and PCL/SBA3_Cu, all the elements forming the filler, such as silicon, sodium, calcium, boron and phosphorous, were detected by spectroscopy. In the PCL/SBA3_Cu sample, small traces of copper (introduced by the ion-exchange process on the powder form) were highlighted, due to the limited amount of copper doped into the BG composition and the amount of filler in weight inside the PCL matrix. The % of the BG elements was well balanced between the two compositions, PCL/SBA3 and PCL/SBA3_Cu, confirming the efficiency of the adopted mixing method [28].

### 3.2. Establishing the ASCs/HMEC-1 Co-Cultures

**Figure 2:**
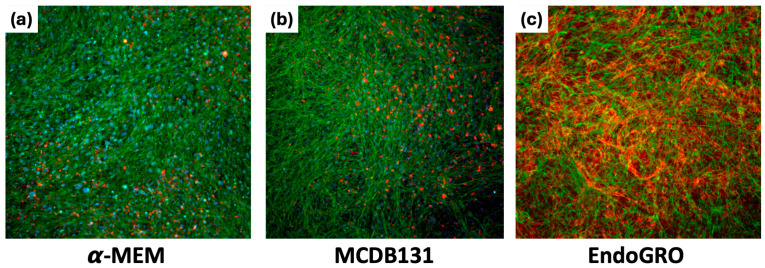
Visualization of ASC/HMEC-1 co-cultures in the different media. Immunofluorescence images at 10× magnification of ASCs co-cultured with HMEC-1 in different media: (**a**) α-MEM, (**b**) MCDB131, and (**c**) EndoGRO. The cytoskeleton is green, CD31 (PECAM) is red, the nuclei are blue.

To investigate what could be the best medium to promote the formation of an effective endothelial network in co-cultures of ASCs and HMEC-1, we tested the maintaining medium of ASCs (α-MEM), the maintaining and the differentiating media of HMEC-1 (MCDB131 and EndoGRO, respectively). The presence of CD31+ endothelial cells was detectable both in α-MEM (Figure 2a) and in MCDB131 (Figure 2b), but a branched, CD31-positive vascular network was achieved only with EndoGRO (Figure 2c), which was the best condition supporting a capillary network formation.

### 3.3. ASC/HMEC-1 Co-Cultures Organize Differently According to the Substrate Materials

**Figure 3:**
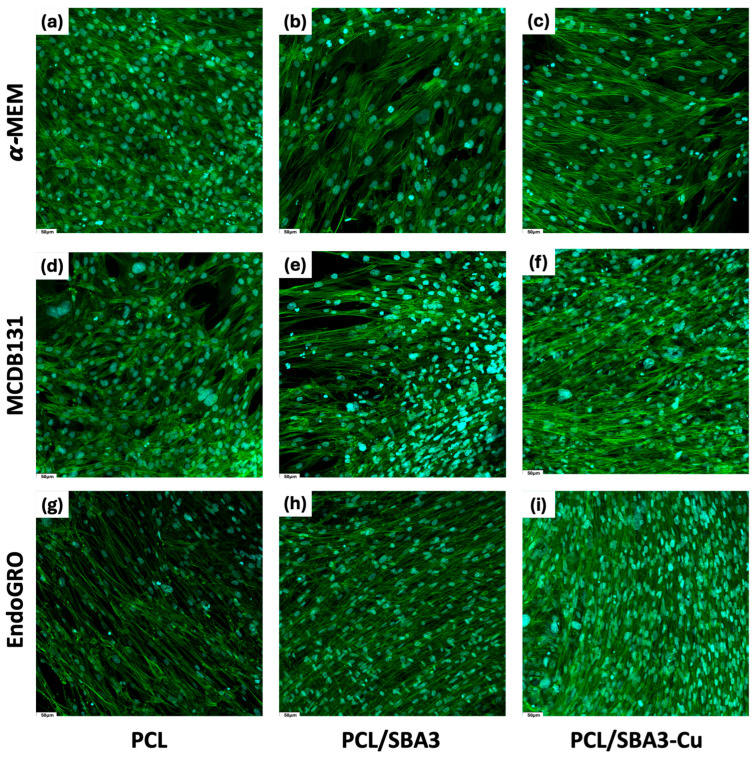
Visualization of ASC/HMEC-1 co-cultures on different materials and in different media. Confocal immunofluorescence images of ASCs and HMEC-1 co-cultured in α-MEM medium, MCDB131 and EndoGRO, respectively, onto PCL (**a**,**d**,**g**), PCL/SBA3 (**b**,**e**,**h**) and PCL/SBA3_Cu (**c**,**f**,**i**). The nuclei are visible in blue, the cytoskeleton in green and CD31 in red. Magnification 20×.

To study whether and how the different materials could affect the growth and the organization of the cellular network made by the co-cultures of ASCs and HMEC-1, cells were stained for CD31 and phalloidin. CD31 was not detectable in any culture condition (Figure 3a–i), while HMEC-1s grown on standard culture plates as a control (alone or co-cultured with ASCs) were positive for CD31 (Supplementary Figure S1). A different pattern of cellular disposition was observed according to the materials and cell culture media, as evident by the green phalloidin staining. In particular, in α-MEM and MCDB131 cells, a more elongated morphology was assumed on PCL/SBA3 (Figure 3b,e) and PCL/SBA3_Cu (Figure 3c,f) than on PCL (Figure 3a–d), forming a fascicular structure. In EndoGRO, the cells showed the typical spindle-shaped morphology, forming bundles on all the materials, with a higher density on PCL/SBA3 (Figure 3h) and PCL/SBA3_Cu (Figure 3i) than on PCL (Figure 3g).

**Figure 4:**
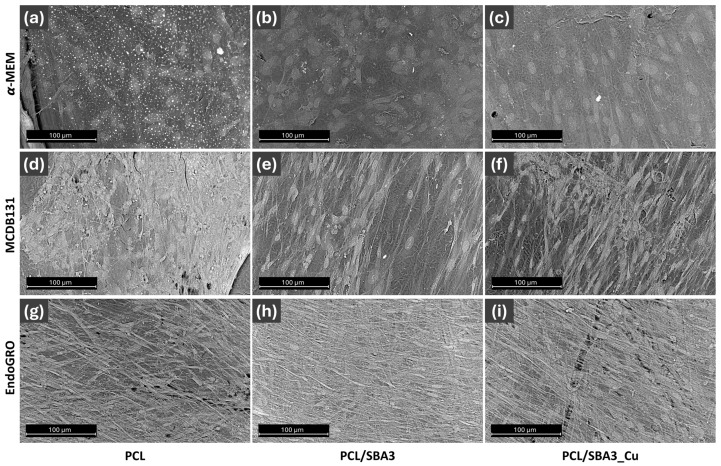
SEM analysis of ACS/HMEC-1 co-cultures. SEM micrographs at 1550× magnification, of co-cultures of ASCs and HMEC-1, on different material substrates, in different culture media and after 21 days. The co-cultures in α-MEM are represented in (**a**) for PCL, (**b**) for PCL/SBA3 and (**c**) for PCL/SBA3_Cu. The co-cultures in MCDB131 are reported in (**d**) for PCL, (**e**) for PCL/SBA3 and (**f**) for PCL/SBA3_Cu. The results from co-cultures in EndoGRO are showed in (**g**) for PCL, (**h**) for PCL/SBA3 and (**i**) for PCL/SBA3_Cu.

The SEM analysis of the cells grown on the surfaces of the different materials confirmed the confocal image visualization. In both α-MEM and MCDB131, more rare and distant cells were present on PCL/SBA3 (Figure 4b,e) and PCL/SBA3_Cu (Figure 4c,f) than on PCL (Figure 4a,d). In EndoGRO, cells grew as a thicker network on PCL/SBA3 (Figure 4h) and PCL/SBA3_Cu (Figure 4i) than on PCL (Figure 4g).

### 3.4. Monitoring the OB-like and Endothelial Cellular Subset on ASC/HMEC-1 Co-Cultures

**Figure 5:**
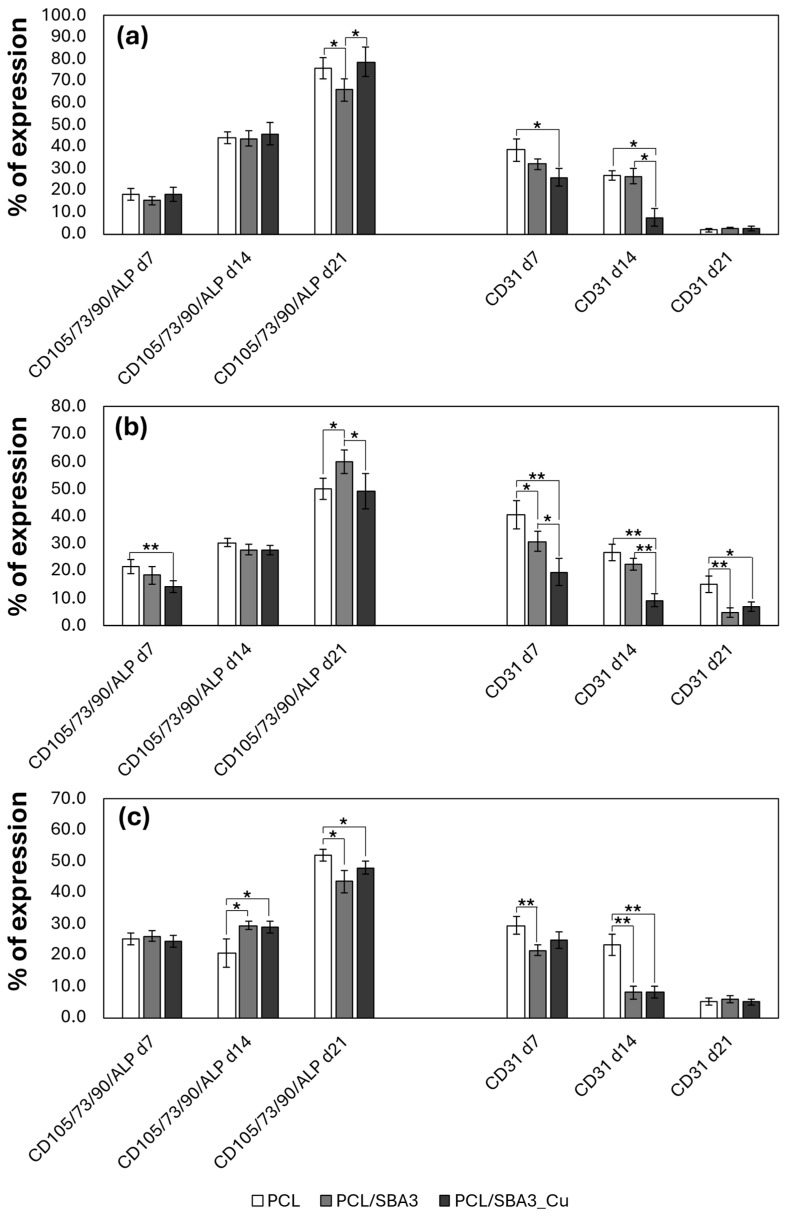
Monitoring the phenotype of ASCs and HMEC-1 in co-culture. The graphs report the % of cells expressing CD105/CD73/CD90/ALP and CD31 in αMEM (**a**), MCDB131 (**b**) and EndoGRO (**c**) at 7, 14 and 21 days of co-culture. * *p* < 0.05, ** *p* < 0.01.

To investigate the possible cause of the missing detection of CD31 signal in 21-day co-cultures, the phenotypes of ASCs and HMEC-1 were monitored by assessing the percentage of the OB-like cellular subset expressing CD105, CD73, CD90 and ALP and the endothelial cells expressing CD31 after 7, 14 and 21 days of culture. On day 7, the % of the OB-like subset was comparable among the different materials in both α-MEM and EndoGRO (Figure 5a,c), while it was reduced on PCL/SBA3_Cu in MCDB131, *p* < 0.05 (Figure 5b). The endothelial cellular subset was significantly higher on PCL than on PCL/SBA3_Cu in α-MEM and MCDB131, *p* < 0.05 and *p* < 0.01, respectively (Figure 5a,b), while, in EndoGRO, CD31+ cells were higher on PCL than on PCL/SBA3, *p* < 0.05 (Figure 5c). On day 14, we observed an increased % of the OB-like subset for all the materials and, in particular, the augmentation was significant on PCL/SBA3 and PCL/SBA3_Cu compared to PCL in EndoGRO, *p* < 0.05. On day 21, a further increase in the OB-like subset was registered for all the materials and media. Conversely, starting from day 14, a decreasing trend was reported for CD31+ cells on both PCL/SBA3 and PCL/SBA3_Cu in all media. On day 21, CD31+ cells were dramatically decreased on all the materials with all the cell media.

### 3.5. Evaluation of the Angiogenic Potential of the PCL/Bioactive Glasses in Different Media

**Figure 6:**
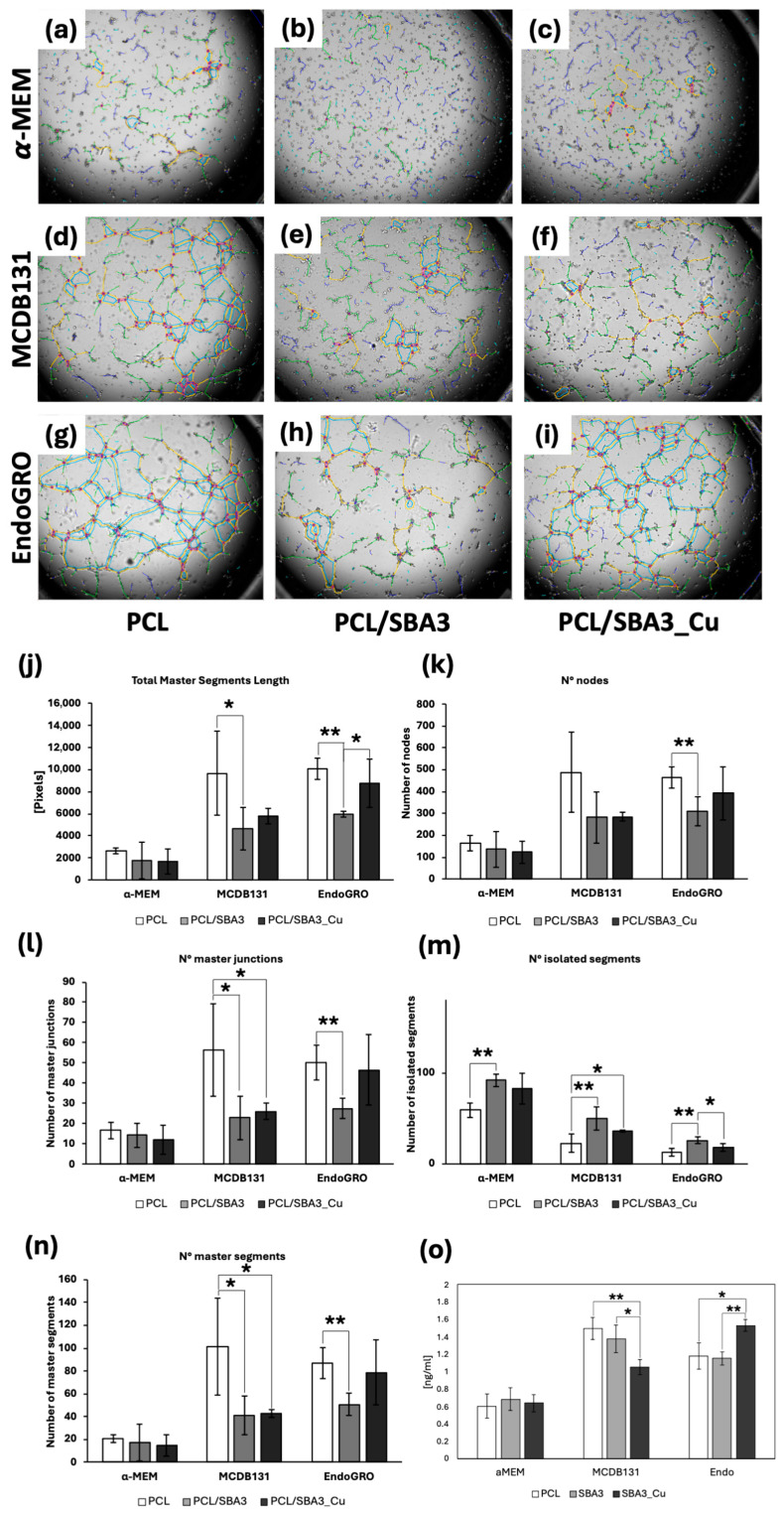
Tubulogenesis assay and VEGF-A quantification. After 15 h, HMEC-1 organization is visible in supernatants of PCL α-MEM (**a**), PCL/SBA3 α-MEM (**b**), PCL/SBA3_Cu α-MEM (**c**), PCL MCDB131 (**d**), PCL/SBA3 MCDB131 (**e**), PCL/SBA3_Cu MCDB131 (**f**), PCL EndoGRO (**g**), PCL/SBA3 EndoGRO (**h**), and PCL/SBA3_Cu EndoGRO (**i**). Graphs indicating the different parameters analyzed in the tubulogenesis assay (**j**–**n**), with * *p* < 0.05 and ** *p* < 0.01. VEGF-A quantification [ng/mL] in the different supernatants (**o**). Segments: magenta; Master segments: yellow; Branches: green; Junctions: red surrounded by blue; Meshes: cyan; Isolated segments: blue.

To assess the pro-angiogenic potential of the different experimental conditions, an in vitro tubulogenesis assay was performed. Results showed that HMEC-1s in α-MEM were unable to form a precise capillary structure, independently of the type of sample, whereas, when plated in MCDB131 and EndoGRO, PCL allowed endothelial cells to form a more organized network, as well as PCL/SBA3_Cu in EndoGRO (Figure 6a–i). To better characterize the maturation and the complexity of the capillary network, different parameters have been considered and analyzed. Precisely, the total segment length highlights the maturation in space of the network (Figure 6j), while the number of nodes, junctions, segments and isolated segments evidences the interconnection inside the network (Figure 6k–n). Taken all together, these parameters showed that BGs did not affect the tubulogenesis of endothelial cells cultured in α-MEM, which, indeed, were not capable of developing a structured network. In MCDB131 medium, tubulogenesis of HMEC-1 was significantly reduced in the presence of both PCL/SBA3 and PCL/SBA3_Cu compared to PCL (Figure 6j,l–n, * *p* < 0.05, ** *p* < 0.01). In EndoGRO medium, PCL/SBA3 did not allow endothelial cells to organize in a complex capillary network, as evidenced by all the evaluated parameters, which were significantly different from PCL in EndoGRO (Figure 6j–n, ** *p* < 0.01). By contrast, PCL/SBA3_Cu in EndoGRO showed tubulogenic capability comparable to PCL, and significantly higher than PCL/SBA3, when considering the maturation of the endothelial network (Figure 6k, * *p* < 0.05). To further elucidate the angiogenic capability of the different experimental settings, the quantification of VEGF-A, an extremely potent pro-angiogenic factor, was performed in the supernatants of the cellular co-cultures (Figure 6o). The co-culture grown on PCL/SBA_Cu released a significant higher level of VEGF-A in EndoGRO compared to the other conditions, but this concentration (1.54 ng/mL) was similar to those quantified in MCDB131 on PCL and PCL/SBA3.

### 3.6. Different Copper Salt Concentrations Affect the Tubulogenic Capability

**Figure 7:**
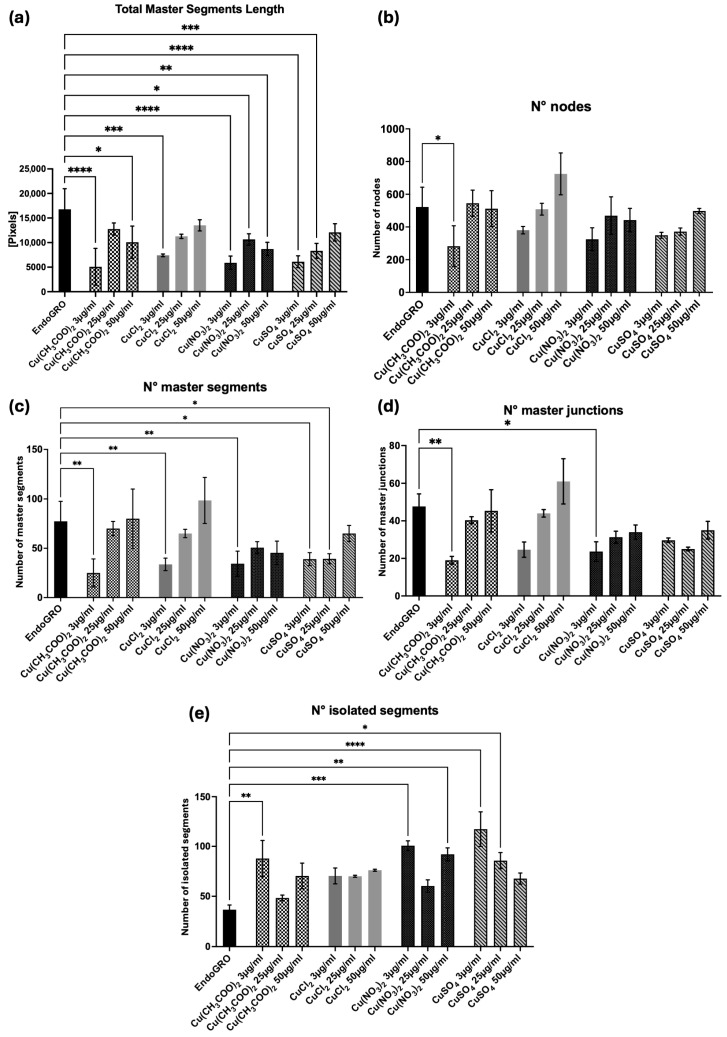
Tubulogenesis assay. (**a**–**e**) Graphs representing the parameters evaluated and analyzed in the assay. * *p* < 0.05, ** *p* < 0.01, *** *p* < 0.001, and **** *p* < 0.0001.

To test the pro-angiogenic potential of Cu^2+^ ions in the in vitro tubulogenesis assay, four copper salts, namely copper acetate [Cu(CH_3_COO)_2_], copper chloride (CuCl_2_), copper nitrate [Cu(NO_3_)_2_], and copper sulfate (CuSO_4_), were tested, at three different concentrations: 3 µg/mL, 25 µg/mL and 50 µg/mL, to understand which one could stimulate tubulogenesis. Although none of the experimental conditions ever outperformed the EndoGRO medium, which was the positive control, it is quite evident that the lowest concentration of copper acetate (3 µg/mL) was always significantly the least effective. In fact, HMEC-1 were not capable of creating an organized and mature capillary network, as shown by all the analyzed parameters (Figure 7a–e), which were significantly different compared to the control. Moreover, copper chloride, copper nitrate, and copper sulfate, even though not significantly, were shown to be less angiogenic at the lowest concentration, which was higher than the concentration of copper released in the media by PCL/SBA3_Cu, reported in previously published work (1.33 ± 0.01 µg/mL after 1 day of incubation in media) [24].

## 4. Discussion

Angiogenesis is essential for bone formation and remodeling, preceding osteogenesis and determining its efficiency during fracture healing. However, the complete vascularization of alloplastic bone grafts is still an unmet task in BTE. Theoretically, the most biomimicking strategy ought to entail biomaterial functionalization with specific physiological pro-angiogenic cues, like vascular endothelial growth factor (VEGF) [29,30]. Unfortunately, this approach is limited by the short half-lives of the growth factors [31,32] and by the potential safety of highly super-physiological concentrations; indeed, VEGF-A can induce aberrant angiogenesis when it is expressed above a threshold level [33]. In addition, growth-factor-based therapies often fall under complex biologics or advanced therapy regulatory frameworks, particularly in Europe, resulting in lengthy approval processes and substantial development costs [34,35]. Several in vitro studies have reported increases in angiogenic indicators owing to the contact of cells either directly with BG particles or indirectly with their dissolution products [36,37,38].

In vivo studies have demonstrated that engineered BG compositions and scaffolds can enhance bone regeneration and vascularization in animal models, including critical-size bone defects in rats and other preclinical models [39,40]; thus, the incorporation of BGs into BTE scaffolds has been regarded unanimously as beneficial in regenerative medicine. Nevertheless, for instance, concentrations of 45S5 Bioglass greater than 0.1% (w = v) were proven to inhibit the secretion of VEGF from fibroblasts, possibly because of the cytotoxicity related to either the augmented ion concentration or the higher pH of the culture medium [41,42,43]. Therefore, understanding the ion dissolution kinetics is mandatory, along with the specific biological effect of each chemical element.

In this research, the authors tested the angiogenic potential of two different BG formulations charged at 10 wt% in PCL, owing to their promising mechanical and biocompatibility features [24]. Both PCL/SBA3 and PCL/SBA3_Cu presented a good dispersion of the BG particles, without any significant filler aggregates. In the PCL/SBA3_Cu sample, small traces of copper were reported by EDX analysis, according to the limited amount of copper inside the bioactive glass composition reported in previous work [25].

The co-culture model used in this work was based on previous observations of the synergistic crosstalk between ASCs and HMEC-1 [44], and it was optimized by selecting EndoGRO as the best medium supporting the formation of an endothelial network in vitro. The same experimental setting was repeated on PCL, PCL/SBA3 and PCL/SBA3_Cu. Unexpectedly, CD31 was not detectable in any culture condition, so cells derived from the co-cultures were detached and analyzed by flow cytometry at different time points after 7, 14 and 21 days. The results from the analyses highlighted that, instead of increasing, the endothelial cellular subset decreased dramatically over time to a small percentage (possibly under the detection threshold of the fluorescent staining), while the OB-like cells increased. A possible explanation of this finding might be the presence of an intrinsic osteogenic potential, exerted by the ions contained and released by the PCL/SBA3 and PCL/SBA3_Cu. Indeed, as reported by several authors [45,46,47], BG ionic dissolution products, in particular calcium, silicon and sodium, stimulate a robust osteoinductive environment by influencing OB gene expression during differentiation. In parallel, Lv et al. [48] showed that composite scaffolds incorporating silicate-based bioceramic particles can trigger endothelial-to-mesenchymal transition (EndMT); this phenomenon offers a plausible explanation for the reduction in the endothelial subset observed in our study. Beyond these biological factors, the lack of detection of CD31 could depend on PCL, which can exhibit significant autofluorescence and light-scattering effects that interfere with fluorescence microscopy, complicating the detection of CD31 in immunofluorescence assays [49,50].

An in vitro tubulogenesis assay was performed to further investigate the angiogenic behavior of the co-cultures in the presence of the different biomaterials. Based on the accurate descriptors of maturation and interconnection of the vessel network induced by the conditioned media, neither PCL/SBA3 nor PCL/SBA3_Cu could promote the development of a structured vascular network in α-MEM and MCDB131 media. In EndoGRO medium, PCL/SBA3 underperformed PCL and PCL/SBA3_Cu that showed similar tubulogenic capability. Accordingly, Moll et al. tested another formulation of BG (1393-B3 BG) doped with copper or zinc, showing that the incorporations of these ions did not improve the angiogenic stimulating properties of the material [51]. Moreover, Wang et al. [52] also failed to find any apparent effect on the tubule formation elicited by the Cu^2+^ releasing composite (PCL:S53P4–Cu1 = 4:1) in comparison to the non-copper-doped scaffold (PCL:S53P4 = 4:1). Furthermore, the co-culture grown on PCL/SBA3_Cu released a significant higher level of VEGF-A compared to the other conditions in EndoGRO, but this concentration (1.54 ng/mL) was similar to those quantified in MCDB131 on PCL and PCL/SBA3, not permitting the emergence of a clear response pattern within the cellular model adopted.

It is noteworthy that, according to Leu and Leach [38], who explored the in vitro tubulogenesis of 45S5 Bioglass within a co-culture of endothelial cells and fibroblasts, the greatest average number of tubules was generated using 1.2 mg 45S5 Bioglass, whereas other masses (0.12, 0.6, and 6 mg) could not enhance tubule formation. This sheds light on the importance of copper dosage in promoting vascularization. Thus, to test the pro-angiogenic potential of Cu^2+^ ions in the in vitro tubulogenesis assay adopted here, four copper salts, namely copper acetate, copper chloride, copper nitrate, and copper sulfate, were utilized, at three different concentrations, according to previously published data testing the copper sulfate [13]. Although none of the experimental conditions ever outperformed the positive control, it is quite evident that the lowest concentration of copper acetate was always significantly the least effective. The lower effectiveness of copper acetate has also been reported in other studies [53], in this case when compared with copper chloride, although at different concentrations. Moreover, copper chloride, copper nitrate, and copper sulfate, even if not significantly, were shown to be less angiogenic at the lowest concentration, which was higher than the concentration of copper released in the media by PCL/SBA3_Cu, as previously reported (1.33 ± 0.01 µg/mL after 1 day of incubation in media) [24]. It is reasonable to hypothesize that the potential angiogenic effect of copper ions is overwhelmed by the osteoinductive power of the other ions released by the glass, present in greater concentrations and not confined to the first superficial layers.

Further research should concentrate on assessing the effect of specific ion dissolution products from BGs and their relative concentration on angiogenesis in proper standard models. The results from such investigations will enable the formulation of optimal bioactive glass compositions to stimulate angiogenesis. In addition, the possible effect of scaffold morphology on neovascularization must be investigated, including porosity, pore size, interconnectivity, and pore orientation. In this context, the morphology of bioactive glass in particulate form deserves consideration to design new BGs with angiogenic potential.

## 5. Conclusions

The angiogenic performance of PCL-based scaffolds incorporating SBA3 and Cu-doped SBA3 BGs was investigated using an ASC/HMEC-1 co-culture model. While both composites displayed homogeneous particle dispersion and good biocompatibility, they did not significantly promote angiogenesis. Analysis performed at different time steps revealed a progressive reduction in the endothelial cell subset accompanied by an increase in osteoblast-like cells, suggesting that the ions released from the bioactive glasses predominantly promote osteogenic differentiation rather than angiogenesis. Although the Cu-doped formulation induced a modest increase in VEGF-A release, the amount of Cu^2+^ released was likely insufficient to elicit a strong pro-angiogenic response, particularly in the presence of other ions with well-established osteoinductive activity. Overall, these findings emphasize the critical role of ion composition and dissolution kinetics in determining the biological response to scaffolds filled with bioactive glass. Future investigation must focus on fine-tuning the ionic release profiles and scaffold architecture to achieve a balanced stimulation of both vascularization and bone formation.

## Figures and Tables

**Table 1:** Formulations of the specimens.

**Specimens**	**PCL [g]**	**SBA3 [g]**	SBA3_Cu [g]
PCL	10	/	/
PCL/SBA3	9	1	/
PCL/SBA3_Cu	9	/	1

## Data Availability

The raw data supporting the conclusions of this article will be made available by the authors on request.

## Supplementary Materials

The following supporting information can be downloaded at: https://www.mdpi.com/article/10.3390/biomedicines14051109/s1, Figure S1: Immunofluorescence images to visualize CD31. Confocal immunofluorescence images of HMEC-1 cultured onto plastic in EndoGRO medium alone (a) and co-cultured with ASCs (b). CD31 is visible in red. Magnification 10×.

## Abbreviations

The following abbreviations are used in this manuscript:

ASCs	Adipose-derived mesenchymal stem/stromal cells ASC52telo
BG	Bioactive glass
BTE	Bone tissue engineering
ELISA	Enzyme-linked immunosorbent assay
EndoGRO	EndoGRO™-MV-VEGF
EndMT	Endothelial to mesenchymal transition
FBS	Fetal bovine serum
FDM	Fused deposition modeling
HA	Hydroxyapatite
HMEC-1	Human microvascular endothelial cells
OB	Osteoblast
PCL	Poly(ε-caprolactone)
PBS	Phosphate-buffered saline
PDGF	Platelet-derived growth factor
SBA3	Silica-based bioactive glass
SBA3_Cu	Silica-based bioactive glass doped formulation
SEM	Scanning electron Microscope
VEGF	Vascular endothelial grow factor
β-TCP	β-tricalcium phosphate
