# Supplementary material for: Assessing the Angiogenic Potential of Poly(ε-Caprolactone) PCL/Bioactive Glass Composites in a Co-Culture Model of ASCs and HMEC-1
Source: Biomedicines. 2026 May 14;14(5):1109. doi: 10.3390/biomedicines14051109 (PMC13204082; doi:10.3390/biomedicines14051109)
Supplement: Supplementary file 1 [file biomedicines-14-01109-s001.zip › biomedicines-4239525-supplementary.pdf]

SUPPLEMENTARY FIGURES

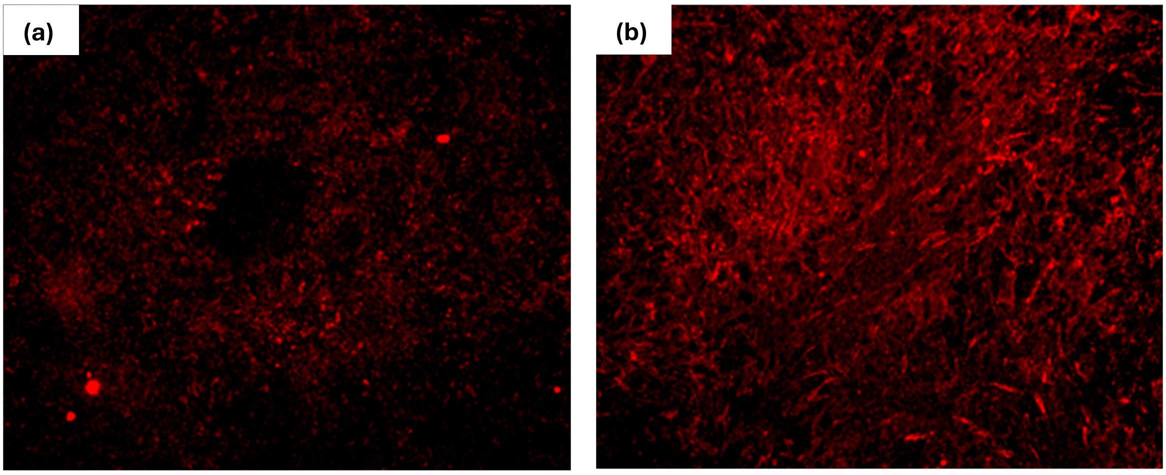

Figure S1. Immunofluorescence images to visualize CD31. Confocal immunofluorescence images of HMEC-1 cultured onto plastic in EndoGRO medium alone (a) and co-cultured with ASCs (b). CD31 is visible in red. Magnification 10×.
